# Molecular mechanisms of plant NLR activation and signalling

**DOI:** 10.1111/tpj.70702

**Published:** 2026-01-30

**Authors:** Natsumi Maruta, Mitchell Sorbello, Laura Garzon‐Flores, Bostjan Kobe

**Affiliations:** ^1^ School of Chemistry and Molecular Biosciences, Institute for Molecular Bioscience and Australian Infectious Diseases Research Centre The University of Queensland Brisbane Queensland 4072 Australia

**Keywords:** coiled‐coil domain, effector‐triggered immunity, nucleotide binding leucine‐rich repeat receptor, plant immunity, resistosome, Toll/interleukin‐1 receptor domain

## Abstract

Plants rely on NLRs (nucleotide‐binding leucine‐rich repeat receptors) to recognise effector proteins secreted by pathogens into plant cells and to deliver disease resistance. Plant NLRs are broadly characterised by their N‐terminal domains, which include the TIR (Toll/interleukin‐1 receptor) and the CC (coiled‐coil) domains. Effector recognition triggers NLR oligomerisation into complexes termed resistosomes, which initiate immune signalling. Some NLRs function as singletons that detect pathogens and activate immune responses, while there are NLRs that only recognise effectors and thereby require helper NLRs or genetically linked ‘paired’ NLRs to execute immune signalling. Recent studies have enhanced our understanding of the molecular mechanisms of different classes of NLRs, as well as how downstream proteins are recruited to signal upon effector recognition. In this review, we discuss the current knowledge of the NLR activation mechanisms, based on findings from recent structural and functional studies. We also highlight the remaining unknowns in the field and discuss current and potential future applications for enhancing plant immunity by engineering plant NLRs.

## Significance Statement

Plant NLRs (nucleotide‐binding leucine‐rich repeat receptors) are essential for recognition of pathogen secreted effectors and activation of effector‐triggered immunity. This review focuses on our current understanding of the NLR activation and signalling mechanisms, based on recent structural and functional studies. It also highlights remaining knowledge gaps in the field, and applications for improving disease resistance by NLR engineering approaches.

## INTRODUCTION

Plants face constant threats from diverse pathogens causing diseases. To defend against pathogens, plants have evolved an innate immune system involving two distinct types of receptors. Cell‐surface PRRs (pattern recognition receptors) detect PAMPs (pathogen‐associated molecular patterns), leading to PTI (PAMP‐triggered immunity). However, host‐adapted pathogens have evolved to secrete effector proteins into plant cells, suppressing PTI and plant development and promoting disease susceptibility. To counter this, plants have evolved intracellular NLRs (nucleotide‐binding leucine‐rich repeat receptors) that can directly or indirectly recognise pathogen effectors and activate ETI (effector‐triggered immunity), which is a robust immune response characterised by localised cell death referred to as the HR (hypersensitive response) (Jones & Dangl, 2006). Immune signalling activated by PRRs and NLRs converges, with these pathways potentiating each other to achieve complete immune responses and restrict pathogen spread (Ngou et al., 2022).

Plant NLRs typically consist of a tripartite domain architecture: a C‐terminal LRR (leucine‐rich repeat) domain, a central NB‐ARC (nucleotide‐binding domain shared by Apaf‐1, plant R proteins and CED‐4) domain (Jones et al., 2016) and an N‐terminal signalling domain. The LRR domain is typically involved in directly interacting with its cognate effector or monitoring effector‐mediated modification of a host target or a decoy protein, whereas the NB‐ARC domain, consisting of NBD (nucleotide‐binding domain), HD1 (helical domain 1) and WHD (winged‐helix domain), functions as a molecular switch; it binds either ADP (adenosine diphosphate) or ATP (adenosine triphosphate) to stabilise the inactive and active states, respectively. Effector recognition induces oligomerisation of NLRs through the NB‐ARC domain into multimeric complexes termed resistosomes. In flowering plants, the N‐terminal domain can be a TIR (Toll/interleukin‐1 receptor) or CC (coiled‐coil) and is required for the activation of immune signalling and HR (Maruta et al., 2022). Consequently, NLRs are generally classified as TNLs (TIR‐NLRs) or CNLs (CC‐NLRs), based on their N‐terminal domain. Besides prototypical NLRs, some NLRs contain additional domains involved in effector recognition, including integrated decoy domains, such as the WRKY transcription factor domain in the Arabidopsis TNL RRS1 (resistance to *Ralstonia solanacearum* 1) (Le Roux et al., 2015; Sarris et al., 2015); the HMA (heavy‐metal associated) domain in rice CNLs, such as RGA5 and Pik‐1 (Césari et al., 2014); and the C‐JID (C‐terminal jelly‐roll/Ig‐like domain) present in approximately 50% of TNLs (Ma et al., 2020; Martin et al., 2020).

There are multiple ways in which NLRs have evolved to operate. Some NLRs can function as singletons that both detect effectors and elicit immune responses, while others require downstream helper NLRs, RNLs, containing the N‐terminal CC_R_ (resistance to powdery mildew 8‐like CC) domain. There is also a complex network of NLRs described in Asterids, where multiple sensor CNLs and CNL‐type helper NRCs (NLR‐required for cell death) are required for disease resistance (Wu et al., 2017). Furthermore, some NLRs serve as obligatory pairs (paired NLRs), with distinct roles in pathogen detection (sensor NLRs; often carrying an integrated decoy domain) and signalling (executor NLRs).

While NLRs have been well‐studied in angiosperms, recent studies have demonstrated that, despite a small copy number, NLR genes are also found in green algae, such as chlorophytes (Feng et al., 2024) and charophytes (the closest relative of land plants) (Andolfo et al., 2019; Gao et al., 2018; Shao et al., 2019). The origin of NLRs therefore dates back to the common ancestors of green plants, prior to land colonisation. In charophytes and early land plants such as mosses, liverworts and ferns, NLRs with atypical N‐terminal signalling domains exist, including those with an αβ‐hydrolase, a protein kinase, a MAEPL motif‐containing CC, or a mixed lineage kinase domain (Andolfo et al., 2019; Castel et al., 2025; Chia et al., 2024; Feng et al., 2024; Xue et al., 2012). Algal NLRs are capable of activating immune responses when transiently expressed in the dicot *Nicotiana benthamiana*, suggesting the conserved function of NLRs over a long evolutionary history (Chia et al., 2024; Feng et al., 2024). Chia et al. (2024) also showed that conventional (CC and TIR‐type) and atypical N‐terminal domains of NLRs from early land plants, as well as gymnosperms, cause cell death in *N. benthamiana* (Chia et al., 2024). These recent and future studies characterising NLRs from non‐flowering plants should provide valuable insights into the evolutionary history, diversity and function of plant NLRs.

In this review, we focus on recent advances in understanding NLR activation and signalling in angiosperm NLRs. Here, we start with a brief history of plant NLR research and then summarise recent structural and functional studies of plant NLRs and discuss current views on the activation of signalling by different NLR classes.

## BRIEF HISTORY OF PLANT NLR RESEARCH

In the 1940s, Harold Flor studied the interaction between flax and its fungal pathogen flax rust. He identified a ‘gene‐for‐gene’ model of disease resistance because resistance by flax to the pathogen depended on a specific ‘resistance’ gene in the plant and a specific ‘avirulence’ gene in the pathogen (Flor, 1971). When the first resistance genes were cloned, the majority were found to belong to the NLR class of immune receptors and to activate defence responses by recognising avirulence effector proteins delivered by pathogens in the cytoplasm of plant cells (Staskawicz et al., 1984, 1995). The pathogen effectors were subsequently found to be recognised by plant NLRs through direct physical interaction or indirectly (e.g. plant NLRs recognising a modification in the cell caused by the presence of the effector). The direct interaction appears to be easier for the pathogen to escape, leading to an arms race between the pathogen and the host plant, where they continuously evolve new variants of effectors and NLRs in an effort to gain the upper hand (Dodds et al., 2006).

The flax‐flax rust pathosystem also led to some early discoveries of the structure–function relationships responsible for the recognition of specific effectors by plant NLRs (Ellis et al., 1999; Wang et al., 2007). These studies highlighted that recognition depends on surface‐exposed residues in avirulence effectors and the LRR domains in NLRs. Further studies on other pathosystems also implicated other NLR domains in the recognition, in particular ‘integrated domains’.

Until 2019, the understanding of molecular mechanisms of plant NLR function relied substantially on the studies of animal NLRs (Burdett et al., 2019b). It was advances in cryo‐EM (cryogenic‐electron microscopy) techniques that allowed key breakthroughs in understanding how plant NLRs recognise avirulence effectors and how that leads to the formation of active oligomeric resistosome complexes that confer disease resistance (Ma et al., 2020; Martin et al., 2020; Wang, Hu, et al., 2019; Wang, Wang, et al., 2019). The oligomerisation leads the CC domains in CNLs to form Ca^2+^‐permeable channels, facilitating calcium influx and downstream signalling (Bi et al., 2021; Förderer et al., 2022; Wang, Hu, et al., 2019; Zhao et al., 2022). In TNLs, by contrast, the TIR domains assemble in a way that activates their NAD^+^‐cleavage activity (Horsefield et al., 2019; Ma et al., 2020; Martin et al., 2020; Wan et al., 2019). The products resulting from NAD^+^‐cleavage explained how TNLs transduce signals downstream, by relaying them to calcium channel‐forming RNLs (Horsefield et al., 2019; Huang et al., 2022; Jacob et al., 2023; Jia et al., 2022; Ma et al., 2020; Manik et al., 2022; Martin et al., 2020; Wan et al., 2019). Several recent structural studies have further expanded our knowledge on helper NRCs in solanaceous plants (Liu, Yang, et al., 2024; Ma et al., 2024; Madhuprakash et al., 2024; Selvaraj et al., 2024).

## EFFECTOR‐INDUCED ACTIVATION OF CNLs

In 2019, three cryo‐EM structures of Arabidopsis ZAR1 (HopZ‐activated resistance 1), representing its inactive, intermediate and active states, were reported, describing for the first time the structural basis of plant NLR activation mechanism (Burdett et al., 2019a; Wang, Hu, et al., 2019; Wang, Wang, et al., 2019). ZAR1 activates defence via a rather complex, indirect effector recognition mechanism in response to several bacterial pathogen effectors. In the inactive state (Protein Data Bank, PDB: 6J5W), ZAR1 forms a heterodimer with a pseudokinase RKS1 (resistance related kinase 1) through LRR‐mediated interactions, and the complex is stabilised by ADP binding to the NBD (Wang, Wang, et al., 2019). ZAR1 indirectly recognises an effector AvrAC from *Xanthomonas campestris*, which uridylates the host kinase PBL2 (PBS1‐like protein 2). The modified PBL2 (PBL2^UMP^) is then recognised by ZAR1:RKS1 (Wang et al., 2015). The intermediate ZAR1:RKS1:PBL2^UMP^ complex (PDB: 6J5V, 6J5U) reveals interaction between PBL2^UMP^ and the RKS1 activation loop. The ZAR1 intermediate state is nucleotide‐free and shows a movement of the NBD compared to its inactive form, suggesting that ADP was released. Ultimately, subsequent ATP binding (dATP used experimentally as a more efficient ligand than ATP) and oligomerisation result in formation of the active pentameric resistosome (PDB: 6J5T) (Wang, Hu, et al., 2019). The most dramatic structural rearrangement occurs in the CC domain. In the inactive state, the CC domains have a compact four‐helix bundle (Casey et al., 2016; Hao et al., 2013; Wang, Wang, et al., 2019). In the active resistosome, the N‐terminal α1 helix protrudes away from the ring, while the other three helices remain together. This α1 helix movement is presumably responsible for puncturing the cell wall membrane, causing Ca^2+^ influx, to trigger HR (Bi et al., 2021). The N‐terminal ZAR1 α1 helix is defined by the conserved MADA motif (MADAxVSFxVxKLxxLLxxEx), which is found in approximately 20% of CNLs in flowering plants, including NRCs, and is the minimal region required to cause cell death and disease resistance (Adachi et al., 2019).

Structural studies of the activated wheat Sr35 (stem rust resistance 35) (PDB: 7XC2, 7XE0), which is also a MADA motif‐containing CNL, demonstrated the formation of a pentameric resistosome with ATP bound to the NB‐ARC domain (Förderer et al., 2022; Zhao et al., 2022). Unlike ZAR1, Sr35 directly binds its cognate effector AvrSr35 from *Puccinia graminis* f. sp. *tritici* through the C‐terminal region of its LRR domain (Förderer et al., 2022; Zhao et al., 2022). While the α1 helix of the CC domain could not be resolved in the Sr35 resistosome structure, mutations in this region resulted in loss of calcium channel activity in *Xenopus* oocytes, supporting the conserved CNL‐mediated function as shown for ZAR1 (Förderer et al., 2022).

**Figure 1:**
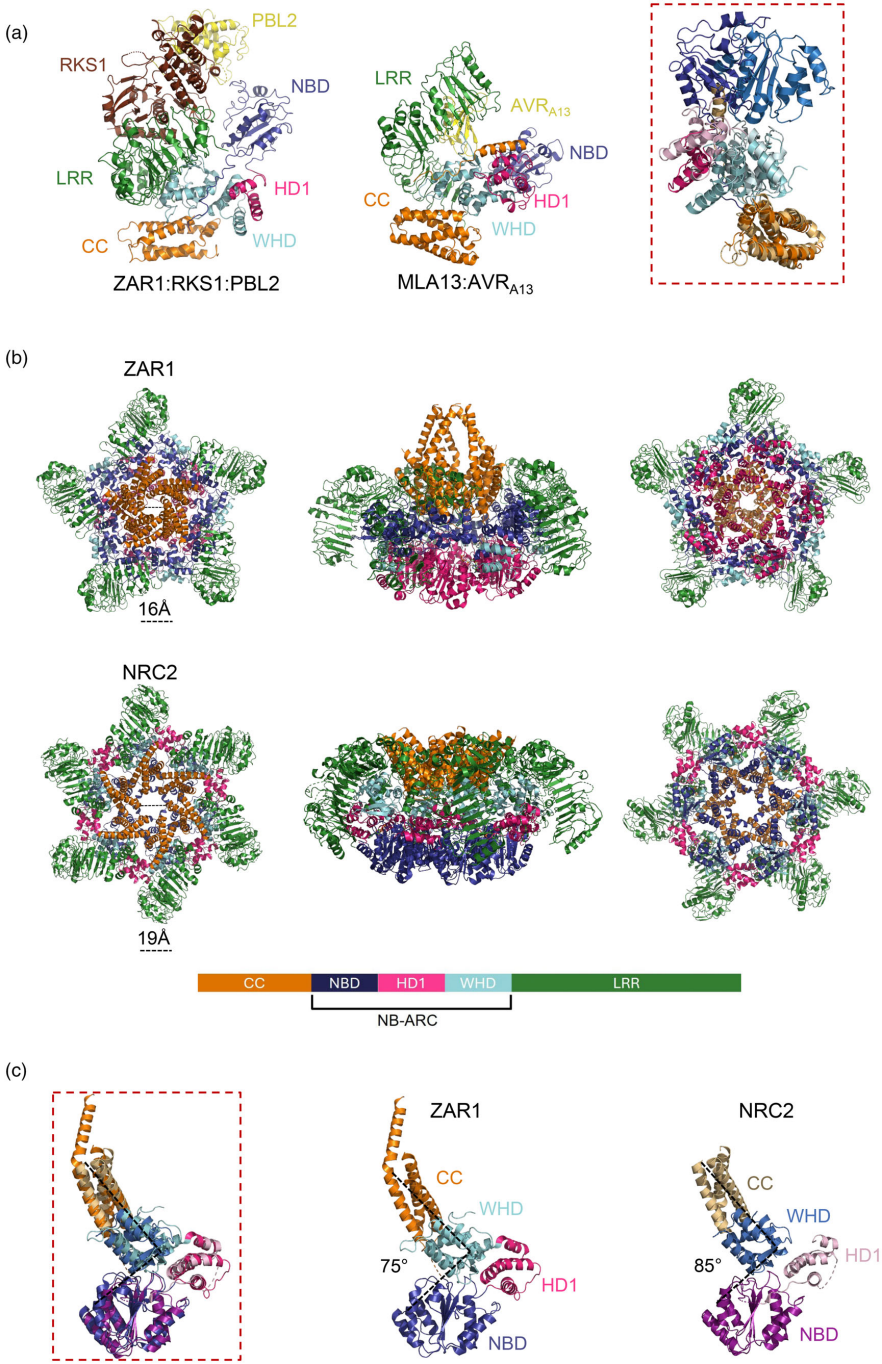
Structural comparison of activated CNLs in different oligomeric states. (a) Domain organisation and structural comparison of ZAR1 and MLA13 CNL proteins. The intermediate state of ZAR1 (left panel; PDB: 6J5V) bound to RKS1 (brown) and PBL2^UMP^ (yellow) exhibits conformational similarities to MLA13 (middle panel; PDB: 9FYC) in complex with AVR_A13_ (yellow). Red box: Structural superposition of NB‐ARC and CC domains of effector‐activated ZAR1 (intermediate state) and MLA13 receptors reveals a difference in positioning of the NBD (purple for ZAR1 and blue for MLA13). Minor positioning differences are observed in the WHD (cyan for ZAR1 and pale blue for MLA13) and HD1 (dark pink for ZAR1 and pale pink for MLA13). The CC domains (orange for ZAR1, pale orange for MLA13) similarly adopt the characteristic four‐helix bundle conformation, which differs from the activated ZAR1 resistosome state. (b) Oligomeric diversity in CNL resistosome architectures. Top row: the ZAR1 pentameric resistosome (PDB: 6J5T; RKS1 and PBL2 omitted) displayed in top, side and bottom views (left to right), showing a five‐fold symmetrical assembly. Middle row: the NbNRC2 hexametric resistosome (PDB: 9FP6) in corresponding orientations, presenting a six‐fold symmetry with tighter inter‐protomer packing compared to ZAR1. Bottom: Colour scheme for domain assignments used throughout the figure: CC (orange), NBD (dark blue), HD1 (pink), WHD (cyan) and LRR (green). (c) Protomer level differences between pentameric and hexameric resistosomes. The red box shows the structural superposition of ZAR1 and NbNRC2 protomers of their resistosomes (PDB: 6J5T and 9FP6; LRRs, RKS1 and PBL2 omitted), highlighting subtle differences in the positioning between the CC domain (orange for ZAR1 and pale orange for NRC2) and the NB‐ARC subdomains. Compared to the ZAR1 protomer (middle panel), the NbNRC2 protomer (right panel) exhibits a ~10° higher angle, which promotes tighter packing and positions the NBD (cyan for ZAR1 and pale blue for NRC2) outwards, allowing for an extra protomer in the activated resistosome.

Contrasting the pentameric resistosomes discussed above, the structure of the MLA13:AVR_A13_‐1 complex revealed a stable heterodimer (Lawson et al., 2025). The barley CNL MLA (mildew locus A) 13 was co‐expressed with its effector protein AVR_A13_‐1 from *Blumeria hordei* in *N. benthamiana*, and the structure of the resulting purified complex was resolved by cryo‐EM (PDB: 9FYC) (Lawson et al., 2025). The heterodimer resembles the ZAR1 intermediate state, based on the relative position of the CC, HD1, WHD and LRR domains (Figure 1a). Like the ZAR1 intermediate state, the cryo‐EM density map shows no evidence for ATP or ADP at the conserved nucleotide‐binding site. However, the NBD of the effector‐bound MLA13 displays conformational flexibility (Figure 1a; right panel). Further analysis showed that the consensus position of the NBD is rotated and positioned further away compared to the ZAR1 intermediate or active form. The CC domain is in its inactive, four‐helix bundle conformation, different from the dimeric crystal structure of the related protein MLA10 determined previously (Casey et al., 2016; Maekawa et al., 2011). The authors present different hypotheses to explain the differences from other effector‐bound CNL structures and the absence of the MLA13 resistosome: (i) the canonical pentameric resistosome falls apart during the purification process; (ii) the pentameric resistosome is present but below the detectable threshold for biophysical or cryo‐EM analysis; or (iii) the heterodimer may represent a novel activated state to induce immune signalling distinct from that mediated by pentameric CNL resistosomes (Lawson et al., 2025). Future studies will be essential to determine the physiological relevance of this heterodimeric structure, to elucidate the precise signalling mechanisms employed by barley MLA proteins in powdery mildew resistance.

## STRUCTURAL BASIS OF ACTIVATION OF HELPER NRCs IN ASTERIDS

NRCs are CNLs containing the N‐terminal MADA motif. NRC2, NRC3 and NRC4 act downstream of various sensor CNLs, as well as PRRs, and exhibit functional redundancy (Kourelis & Adachi, 2022). For example, the sensor CNL, Rx, detects the coat protein (CX) from PVX (potato virus X), resulting in the activation of NRC2, NRC3 and NRC4 (Wu et al., 2017). Similarly, all three NRCs are activated upon sensor CNL Bs2‐dependent recognition of AvrBs2 from *X. campestris*, while only NRC4 contributes to HR upon oomycete AVRblb2‐induced activation of the sensor CNL, Rpi‐blb2 (Wu et al., 2017).

Recent structural studies reveal NRCs in their inactive, active and effector‐inhibited forms. These NRC structures demonstrate similarities and differences compared to singleton CNLs and have substantially expanded our understanding of NRC activation. Unlike ZAR1, which is monomeric in its autoinhibited state (Wang, Hu, et al., 2019), NRC2 from *N. benthamiana* (hereafter NbNRC2) forms a homodimer in its resting state (Selvaraj et al., 2024), and dimeric, tetrameric and filamentous assemblies were also observed for the inactive *Solanum lycopersicum* NRC2, SlNRC2 (Liu, Yang, et al., 2024; Ma et al., 2024). PVX CP‐induced activation of Rx leads to the formation of a homo‐hexameric resistosome by NbNRC2 (Madhuprakash et al., 2024) and SlNRC3 (PDB: 9RI9) (Seager et al., 2025). Similarly, the bacterial effector AvrBs2 is recognised by the sensor NLR, Bs2, which subsequently leads to a homo‐hexameric assembly of NbNRC4 (Liu, Yang, et al., 2024).

Cryo‐EM studies of homodimeric NbNRC2 (PDB: 8RFH) and SlNRC2 (PDB: 8XUO) (Liu, Yang, et al., 2024; Selvaraj et al., 2024) show clear EM density for the LRR domain and the ADP‐bound NB‐ARC domain, but not the CC domain, suggesting its flexibility. Dimerisation occurs through interactions between the NBD of one NRC2 protomer and the LRR domain of the other, with an additional interface formed by the N‐terminal LRR domain of both protomers. *In planta* co‐immunoprecipitation assays demonstrate that NbNRC2 self‐associates but does not interact with other NRC paralogs. Sequence analyses revealed divergence within the dimerisation interface among different NRC clades, suggesting their functional specificity (Selvaraj et al., 2024).

The SlNRC2 homodimers can further self‐associate to form tetramers (PDB: 8XUQ), where the lateral LRR side of one protomer interacts with the CC domain of another protomer (Liu, Yang, et al., 2024). Structure‐guided mutagenesis that disrupted self‐association also promoted CP‐induced HR, suggesting that dimerisation and tetramerisation of inactive states are necessary to prevent unwanted immune activation. A filamentous SlNRC2 structure (PDB: 8XUV) consisting of dimeric inactive proteins was also resolved by cryo‐EM, though its physiological relevance remains unclear. Unexpectedly, each SlNRC2 structure revealed the small molecule IP (inositol phosphate) binding between the WHD and the LRR domain (Liu, Yang, et al., 2024). Mutations in the conserved IP‐interacting residues disrupted IP binding and HR. Hence, IPs, known to be involved in various eukaryotic cellular processes, may serve as cofactors modulating SlNRC2 function. However, no IP binding was detected in other NRC structures, and further studies are required to establish their potential roles.

Cryo‐EM 3D reconstructions revealed high‐resolution structures of CP/Rx‐induced activated NbNRC2 (PDB: 9FP6) and an autoactive variant of NbNRC4 (PDB: 9CC8), both forming homo‐hexameric resistosomes (Liu, Yang, et al., 2024; Madhuprakash et al., 2024). Additionally, negative‐stain EM 2D class averages of NbNRC0 and NbNRC3 confirmed their hexameric assemblies (Liu, Yang, et al., 2024), suggesting that hexamer formation is a conserved feature of NRCs. In these cryo‐EM structures, clear EM density was observed for an ATP molecule interacting with the NBD of activated NRCs, resembling Sr35 (Förderer et al., 2022; Li et al., 2022).

So far, there is no evidence for hetero‐oligomer assembly between sensor NLRs and helper NRCs (Selvaraj et al., 2024). Biochemical and functional studies support an activation‐and‐release model, in which effector recognition by a sensor NLR induces a conformational change that activates downstream helper NRCs. These NRCs then form plasma membrane‐localised resistosomes without the incorporation of the sensor NLR (Contreras et al., 2024). Recent BN‐PAGE (blue native polyacrylamide gel electrophoresis) analysis also showed that incubation of purified NbNRC2 with the NBD of Rx and ATP enhanced formation of the NbNRC2 homo‐hexameric resistosome (Selvaraj et al., 2024), consistent with previous findings that the NBD of a sensor NLR is sufficient to activate HR (Rairdan et al., 2008), without incorporation of the sensor NLRs into the activated complex (Contreras et al., 2024). It remains unclear how different sensor NLRs can induce NbNRC2 assembly.

Recently, additional structural studies have provided further insights into how pathogen effectors could evolve to directly target NRCs during activation, and how NRCs may transition from inactive to active states. The RXLR‐LWY effector AVRcap1b from the potato oomycete pathogen *Phytophthora infestans* was found to suppress plant defence by binding multiple host targets, including NRC2 and NRC3 (Seager et al., 2025). Its crystal structure (PDB: 9RDC) revealed a unique L‐shaped fold. In parallel, a recent preprint reports the cryo‐EM structures of AVRcap1b‐bound SlNRC3 in its oligomer intermediate form (PDB: 9RIA) and the SlNRC3 hexameric resistosome (PDB: 9RI9) (Seager et al., 2025). In the intermediate form, three assembled NRC3 protomers are wrapped by AVRcap1b, forming a large interface involving the CC, NBD and WHD domains, thereby inhibiting hexamer formation. In NRCs, the conserved YEFF (Tyr‐Glu‐Phe‐Phe) residues on the NBD periphery are necessary for the interaction with AVRcap1b and cell‐death inhibition. Overall, the *Phytophthora* effector suppresses plant immune signalling by directly interfering with NRC oligomerisation and activation (Seager et al., 2025). Using a truncated AVRcap1b variant as a tool, a tetrameric intermediate state of NRC3 was also captured, suggesting that the NRC resistosome formation likely occurs in a stepwise manner from dissociated monomers.

## STRUCTURAL DIVERSITY AMONG CNL RESISTOSOMES

Both singleton CNL and helper NRC resistosomes show conserved packing and stabilisation of assembled protomers (Förderer et al., 2022; Li et al., 2022; Liu, Yang, et al., 2024; Madhuprakash et al., 2024; Wang, Hu, et al., 2019; Figure 1b). First, like ZAR1 and Sr35, NRC packing requires inter‐protomer interactions of the following: (i) the CC‐CC domain interaction between adjacent protomers, (ii) the HD1 domain of one protomer interacting with the NBD, WHD and LRR domain of another and (iii) the NBD‐NBD contact between adjacent protomers (Liu, Yang, et al., 2024, Madhuprakash et al., 2024). Second, NRCs stabilise the resistosome through intra‐protomer interactions between the EDVID (Glu‐Asp‐Val‐Ile‐Asp) motif in the α3 helix of CC domains and the N‐terminal LRR region containing arginine residues (the R‐cluster motif), forming multiple hydrogen bonds and salt bridges (Liu, Yang, et al., 2024; Madhuprakash et al., 2024). The number of residues involved in this interaction varies. For example, NbNRC2 contains an extended region following the conserved EDVID motif that interacts with the R‐cluster motif, whereas NbNRC4 lacks this extension and relies on only two arginine residues in the R‐cluster.

Despite the similarities mentioned above, the fundamental difference between singleton CNL and helper NRC resistosomes is the stoichiometry (Figure 1b). Detailed structural comparisons revealed key factors explaining this difference (Madhuprakash et al., 2024). First, the angle between the CC and NB‐ARC subdomains of a NbNRC2 protomer is ~10° higher than that of ZAR1 (Figure 1b,c). This orientation positions the NBD of NbNRC2 further outwards, allowing accommodation of an extra protomer. Additionally, compared to ZAR1, the HD1 of one NbNRC2 protomer makes more extensive contacts with a concave surface of an adjacent protomer formed by its NBD, WHD and LRR domains, thereby enabling tight hexamer packing. Finally, the end of the α4 helix of the CC domain in NbNRC2 and NbNRC4 has a kink, whereas the α4 helix of ZAR1 is straight and elongated and that of Sr35 includes a loop extension that would sterically clash with an adjacent protomer in a hexameric assembly (Liu, Yang, et al., 2024; Madhuprakash et al., 2024; Wang, Hu, et al., 2019).

The pores formed by the CC and NB domains in the NRC resistosomes are larger than those in singleton CNL resistosomes (Liu, Yang, et al., 2024). Both NbNRC2 and NbNRC4 CC pores have similar diameters that are 7 Å and 2 Å wider than those of ZAR1 and Sr35, respectively. NB pores formed by NbNRC4 are the widest (33 Å), followed by NbNRC2 (19 Å), ZAR1 (16 Å) and Sr35 (16 Å) (Figure 1b). Different pore sizes may reflect variability in channel activity among CNLs that should be explored in future studies. Transient expression of autoactive NbNRC4 and NbNRC3 variants triggered elevated calcium influx in *N. benthamiana*, but failed to do so in HEK (human embryonic kidney) 293 cells and *Xenopus* oocytes (Liu, Yang, et al., 2024). Also, no plasma membrane localisation was observed in HEK293 cells. These observations differ from ZAR1 and Sr35, which did conduct calcium in *Xenopus* oocytes (Barragan & Weigel, 2021; Förderer et al., 2022), and from NRG1.1, a different class of helper NLR, which localises to the plasma membrane and induces calcium influx in human HEK293 and HeLa cells (Jacob et al., 2021). Altogether, the findings suggest that NRCs may require plant‐derived cofactors for proper subcellular localisation and/or channel activity.

CNLs are classified into sub‐groups, including CC‐NLRs, CC_R_‐NLRs (RNLs) and CC_G10_‐NLRs (G10‐type CNLs) (Kourelis et al., 2021). Detailed mechanisms of how helper RNLs operate downstream of TNLs will be described in the following sections, but structures of activated RNL resistosomes are yet to be elucidated. CC_G10_‐NLRs, such as Arabidopsis RPS2 (resistance to *Pseudomonas syringae* 2) and RPS5, deviate substantially from typical CNLs, with the characteristic EDVID motif in the CC domain being absent (Guo et al., 2025; Lee et al., 2021). A recent preprint article revealed that CC_G10_‐NLR resistosomes formed by RPS2 and WAI3 (wheat autoimmunity 3) both displayed a novel octameric configuration, suggesting a conserved oligomeric state in monocots and dicots (Guo et al., 2025). The WAI3 resistosome cryo‐EM structure shows that unlike ZAR1, Sr35 and NRCs, the CC:LRR intra‐protomer interaction is absent and its CC_G10_ domains are positioned directly on top of NBDs. This unique assembly is supported by the lack of the EDVID motif and the R‐cluster in its LRR domain. Similar to CC‐NLRs and CC_R_‐NLRs, activated CC_G10_‐NLRs can induce robust calcium influx. Overall, different subfamilies of CNLs form calcium channels upon effector recognition, establishing Ca^2+^ signalling as a convergence point in NLR activation.

## TNLs FORM TETRAMERIC RESISTOSOMES

**Figure 2:**
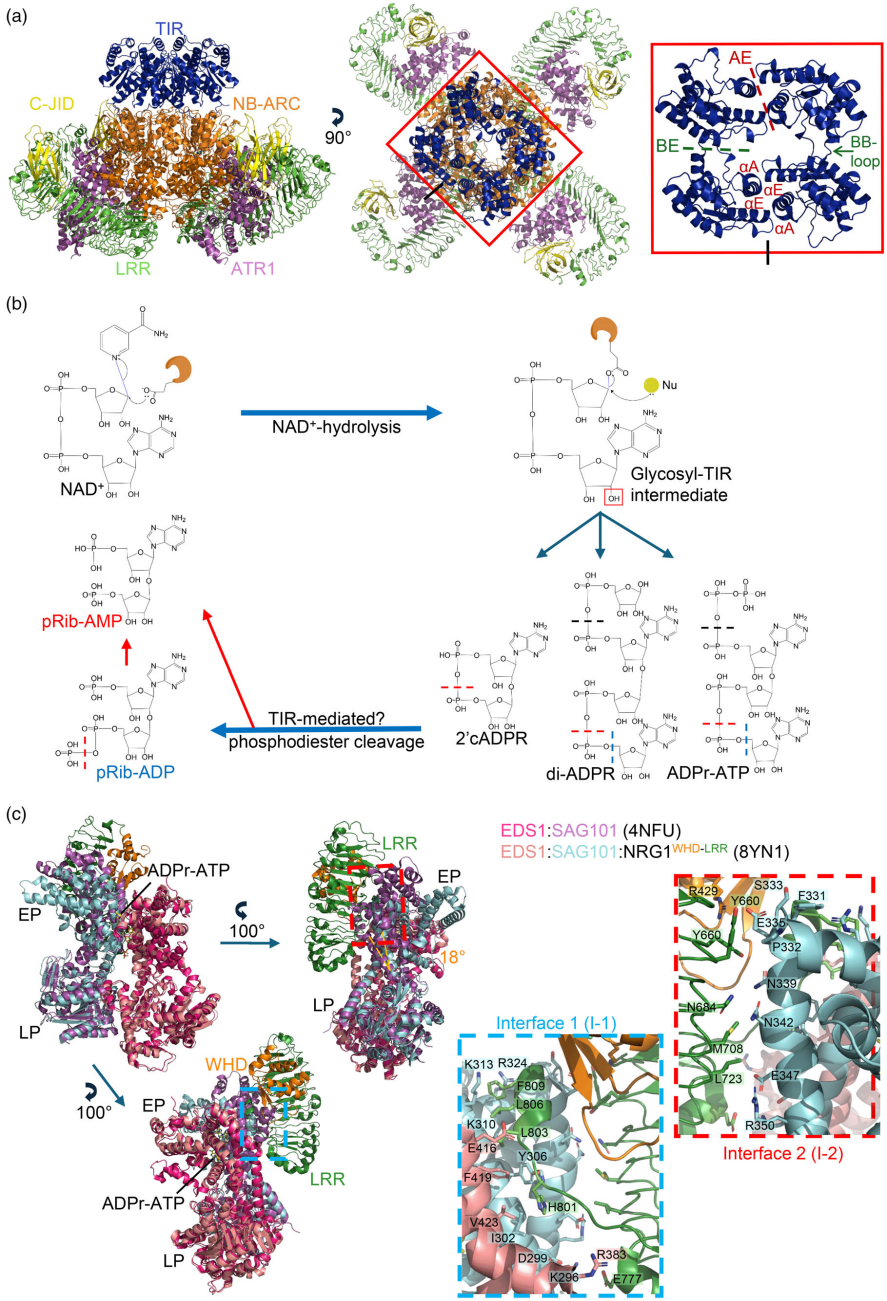
Structural and molecular basis for TNL‐mediated activation of EDS1 family proteins and helper RNLs. (a) The Arabidopsis TNL RPP1 (PDB: 7CRC) resistosome is shown as side and top views. Each domain is coloured as follows: TIR—Toll/interleukin‐1 receptor (blue), NB‐ARC—nucleotide‐binding domain shared by Apaf‐1, plant R proteins and CED‐4 (orange), LRR—leucine‐rich repeat (green) and C‐JID—C‐terminal jelly‐roll/Ig‐like domain (yellow). The pathogen effector ATR1 is shown in purple. The red box highlights the TIR‐domain assembly (middle panel) and is shown as a magnified top view with other domains hidden (right panel). The TIR‐domain interactions through the AE (αA and αE helices from each contacting TIR molecule) and BE interfaces (formed between the αD‐αE surface of a TIR domain and the BB loop of another) mediating TIR‐domain assembly are indicated. The figure was generated using PyMOL. (b) Mechanism of NAD^+^ hydrolysis. The catalytic glutamate binds to the carbon attached to the nicotinamide, which acts as the leaving group. The anomeric carbon undergoes nucleophilic attack, releasing the glutamate. The red box highlights the hydroxyl group (OH) that can act as the nucleophile in a cyclisation reaction to generate 2’cADPR. The same OH group, from another ADPR molecule or an ATP molecule, can also act as a nucleophile in an ADP‐ribosylation reaction to form di‐ADPR or ADPr‐ATP. Phosphodiester cleavage of 2’cADPR, di‐ADPR or ADPr‐ATP could lead to pRib‐ADP (blue dashes) or pRib‐AMP (red dashes). Black dashes represent cleavage sites common to both pRib‐AMP and pRib‐ADP formation. (c) Superposition of the inactive EDS1:SAG101 heterodimeric complex (PDB: 4NFU) onto the EDS1:SAG101:NRG1A^WHD‐LRR^ heterotrimer complex (PDB: 8YN1). The inactive EDS1 from 4NFU (magenta) shows no major conformational differences compared to the activated EDS1 from 8YN1 (salmon). A 100° rotation in both directions along the *y*‐axis is shown. The structure on the top right shows an 18° movement of the EP domain of SAG101. SAG101 from the inactive complex (purple) clashes with the heterotrimer interfaces. Blue and red boxes show magnified views of interface‐1 (I‐1) and interface‐2 (I‐2), with sidechains of relevant residues shown in stick representation. Residues are labelled with shaded boxes. Inactive EDS1:SAG101 (PDB: 4NFU) is hidden in the magnified images. Green—NRG1^LRR^, orange NRG1^WHD^, cyan—SAG101 and salmon—EDS1. EP—EDS1:PAD4 domain, LP—lipase‐like domain.

Cryo‐EM structures of the activated, effector‐bound TNLs ROQ1 (recognition of XopQ 1; from *N. benthamiana*) and RPP1 (recognition of *Peronospora parasitica* 1; from Arabidopsis) demonstrate a conserved mechanism of tetrameric resistosome assembly (Ma et al., 2020; Martin et al., 2020). Following their LRR domains, both TNLs contain extended C‐JIDs that make key contacts with their respective effectors. TIR domains share a conserved tertiary structure comprising a five beta‐strand core (βA‐βE) surrounded by five alpha‐helices (αA‐αE) (Ve et al., 2015). In TNL resistosomes, the NBD‐mediated oligomerisation enables the TIR domains to self‐associate through two interfaces: the symmetric AE (αA and αE helices) interface and the asymmetric BE (BB loop and DE surface) interface (Figure 2a). The BB loop (between the αB helix and βB strand) of one TIR domain is stabilised through interactions with the DE surface of an adjacent TIR domain (residues in αD and αE helices), which allows substrate binding for NAD^+^‐cleavage activity (Bhatt et al., 2024; Ma et al., 2020; Martin et al., 2020; Maruta, Sorbello, et al., 2023; Nimma et al., 2021).

The AE interface is critical for TIR‐mediated signalling, despite not being part of the active site, as mutations in this interface completely abolish cell‐death signalling (Bernoux et al., 2011; Chan et al., 2009; Ma et al., 2020; Martin et al., 2020; Mestre & Baulcombe, 2005; Williams et al., 2014, 2016; Zhang, Bernoux, et al., 2017). A preprint has provided a plausible explanation: during formation of the AE interface, rearrangement of several key residues occurs and promotes opening of the BB loop, and therefore, the AE interface configures the substrate binding pocket (Burdett et al., 2021).

## FORMATION OF HETERO‐OLIGOMERIC COMPLEXES BY PAIRED TNLs

There are NLRs, encoded in genomes in a head‐to‐head orientation, that function as genetically linked pairs, consisting of a sensor NLR responsible for effector recognition and an executor NLR responsible for immune signalling (Schulze et al., 2022). Recent studies provide evidence for hetero‐oligomerisation of the paired TNLs. For example, co‐expression of the Arabidopsis TNL pair, CHS3 (sensor; chilling sensitive 3) and CSA1 (executor; constitutive shade avoidance 1), leads to the formation of a hetero‐oligomer with the size of ~720 kDa (Yang et al., 2024). Detailed analyses of CHS3:CSA1 complexes from two Arabidopsis ecotypes, by BN‐PAGE, co‐immunoprecipitation and cell‐death assays, revealed clade‐specific TIR requirements for assembly and function and suggested their potentially different hetero‐oligomer formation. In the resting state, the CHS3:CSA1 pair interacts with negative regulators, BAK1 (brassinosteroid insensitive 1 associated receptor kinase 1) and BIR3 (BAK1 interacting receptor‐like kinase 3) (Schulze et al., 2022), likely as a heterodimer (Yang et al., 2024). Pathogen‐mediated de‐repression of BAK1 and BIR3 then triggers CHS3:CSA1 oligomerisation, requiring the TIR‐domain AE and BE interfaces (Schulze et al., 2022; Yang et al., 2024). Curiously, another TNL pair, consisting of Arabidopsis RPS4 (executor; resistant to *Pseudomonas syringae* 4) and RRS1 (sensor), forms a stable hetero‐tetramer even in the absence of effectors (Ahn et al., 2025). It is hypothesised that effector recognition induces a structural rearrangement in the TIR domains, leading to enzymatic activity, rather than oligomerisation‐dependent activation. Paired NLRs can have additional functional complexity. The Arabidopsis RPP2 locus encodes up to four TNLs (RPP2a‐d), with RPP2a paired with RPP2b and RPP2c paired with RPP2d (de Weyer et al., 2019; Kim et al., 2024). For full quantitative resistance to *Hyaloperonspora arabidopsidis*, both TNL pairs are required (Kim et al., 2024), using a yet unknown mechanism. Further studies are necessary to structurally and functionally characterise different paired TNLs.

## TIR‐DERIVED SIGNALLING PRODUCTS

Across the domains of life, multiple small molecules generated upon NADase activity by TIR domains have been described (Bayless et al., 2023; Horsefield et al., 2019; Manik et al., 2022; Wan et al., 2019). In Figure 2b, we show the typical NAD^+^ hydrolysis (NADase) reaction, which yields Nam (nicotinamide) and ADPR (adenosine diphosphate ribose) (Horsefield et al., 2019; Shi et al., 2022; Wan et al., 2019). Shi et al. (2022) described the likely molecular basis for this reaction: a glutamate residue containing a carboxylate oxygen forms a covalent linkage to the anomeric carbon in NAD^+^, which is attached to the Nam moiety (Shi et al., 2022). Nam is released, and the anomeric carbon undergoes nucleophilic attack to generate the final product, releasing the glutamate. In a typical hydrolysis reaction, the nucleophile is a hydroxyl group from a water molecule. Interestingly, other compounds can bind to form ‘adducts’ (Manik et al., 2022; Shi et al., 2022). In bacteria, TIR domains from the Thoeris antiphage defence system can use histidine and imidazole to form HAD (histidine‐ADPR) and IAD (imidazole‐ADPR) (Sabonis et al., 2025; Shi et al., 2024). In plant TIRs, ADPR or ATP can be used to produce di‐ADPR (ADPr‐ADPR) and ADPr‐ATP (Jia et al., 2022).

Instead of forming adducts, the anomeric carbon can also cyclise to form cADPR, with four different structural isomers described. The canonical cADPR and the newly discovered N7‐cADPR cyclise through N‐glycosidic linkages, whereas the ones more relevant to plant systems, 2’cADPR and 3’cADPR, cyclise through O‐glycosidic linkages (Bayless et al., 2023; Essuman et al., 2018; Manik et al., 2022; Rousset et al., 2025; Wan et al., 2019). In bacteria, cADPRs and HAD act as secondary messengers that bind and activate downstream proteins (Ofir et al., 2021; Rousset et al., 2025; Sabonis et al., 2025; Shi et al., 2024). Plant TIR domains typically produce 2’cADPR, which could be related to Ca^2+^ influx, as reviewed previously (Maruta, Sorbello, et al., 2023). Additionally, 2’cADPR is a plausible precursor of pRib‐AMP (phosphoribosyl‐adenosine monophosphate; Figure 2b) (Yu, Xu, et al., 2024). pRib‐AMP and pRib‐ADP are plant TIR‐derived molecules predicted to be produced through phosphodiester cleavage of ADPr‐ATP, di‐ADPR and 2’cADPR (Figure 2b; Huang et al., 2022; Yu, Xu, et al., 2024). It remains unknown whether this conversion is regulated solely by TIR domains or involves other enzymes. In summary, plant TIR domains possess NADase activity, where the Nam moiety is cleaved and a glycosyl‐enzyme intermediate is formed. The enzyme is released when the anomeric carbon undergoes nucleophilic attack, either by a hydroxyl group from water, through cyclisation or through adduct formation with other nucleophiles, such as ADPR or ATP.

## TNL s REQUIRE DOWNSTREAM EDS1 PROTEINS AND HELPER RNL‐TYPE NLR s

Activated TNLs relay signals to downstream conserved components: (i) the EDS1 (enhanced disease susceptibility 1) family proteins; and (ii) helper NLRs (RNLs), including NRG1 (N requirement gene 1) and ADR1 (activated disease resistance 1), which act as Ca^2+^ channels (Chen et al., 2024; Feys et al., 2001; Jacob et al., 2021; Lapin et al., 2019; Parker et al., 1996; Sun et al., 2021; Wagner et al., 2013; Wu et al., 2021). The EDS1 family members include EDS1, SAG101 (senescence‐associated gene 101) and PAD4 (phytoalexin deficient 4), and each possesses an N‐terminal LP (lipase‐like) domain and a C‐terminal EP (EDS1‐PAD4) domain. EDS1 forms a heterodimer with SAG101 or PAD4 to recruit NRG1 or ADR1, respectively (Lapin et al., 2020), where association is mediated by the EP domains of EDS1 heterodimers and the LRR domain of RNLs (Chen et al., 2024; Huang et al., 2025; Sun et al., 2021; Wu et al., 2021; Wu et al., 2024; Xiao et al., 2025; Yu, Xu, et al., 2024). Recent studies describe how the EPA (EDS1:PAD4:ADR1) and ESN (EDS1:SAG101:NRG1) modules are activated by TIR domains.

## TIR NAD ase‐DERIVED COMPOUNDS BIND EDS1 HETERODIMERS TO RECRUIT RNL s

pRib‐AMP and pRib‐ADP were discovered to directly activate the EDS1:PAD4 heterodimer (Huang et al., 2022). Comparison of the ligand‐free EDS1:PAD4 cryo‐EM structure (PDB: 7XDD) and the pRib‐ADP‐bound EDS1:PAD4 crystal structure (PDB: 7XEY) revealed that upon pRib‐ADP binding at the pocket formed by EP domains of EDS1 and PAD4, the EP domain of PAD4 undergoes ~20° rotation (Figure 2c; Huang et al., 2022, Yu, Xu, et al., 2024). It was hypothesised that this rotation enables interaction and activation of ADR1 (Huang et al., 2022). The EDS1:SAG101 cryo‐EM structure bound to ADPr‐ATP was also resolved (PDB: 7XJP) and showed ~15° EP rotation (Jia et al., 2022; Wagner et al., 2013). The residues in EDS1:SAG101 that bind to the extended ATP moiety of ADPr‐ATP (or ADPR in di‐ADPR) are substituted with bulky residues in PAD4, which would presumably block binding. In summary, EDS1:PAD4 selectively binds pRib‐ADP and pRib‐AMP, while EDS1:SAG101 selectively binds ADPr‐ATP and di‐ADPR.

## CRYO‐EM STRUCTURES OF EPA AND ESN HETEROTRIMERS

There are no TNLs in monocots, but both monocots and dicots have TIR‐only and EDS1‐family proteins. Two recent studies reported highly similar cryo‐EM structures of rice and Arabidopsis EPA, bound to pRib‐ADP (PDB: 8ZF0 and 8ZW9; pRib‐ADP resulted from NADase activity by a rice TIR‐only protein, OsTIR (Wu et al., 2024) and the TIR domain of Arabidopsis TNL RPS4 (Yu, Xu, et al., 2024), respectively). In the heterotrimer, the EDS1:PAD4 portion is structurally identical to the ADR1‐free, pRib‐ADP‐bound EDS1:PAD4 heterodimer (Huang et al., 2022). The heterotrimer is held together by two interfaces. I‐1 (interface‐1) consists of the EP domains in EDS1 and PAD4 and the LRR domain of ADR1. The larger I‐2 (interface‐2) interactions are mediated almost entirely through PAD4 and ADR1 (Wu et al., 2024; Yu, Xu, et al., 2024). Yu, Xu, et al. (2024) further showed that bacterial TIR domains that produce 2’cADPR, but not 3’cADPR, or direct application of 2’cADPR to plant lysate, can induce pRib‐AMP bound EPA complexes. These observations suggest that 2’cADPR is a plausible precursor to pRib‐AMP. Overall, the EPA module is conserved in both monocots and dicots, and pRib‐AMP and pRib‐ADP derived from either TIR‐only or TNL proteins can serve as second messengers (Huang et al., 2022; Wu et al., 2024; Yu, Xu, et al., 2024).

Similar to the EPA findings, the Arabidopsis ESN heterotrimer structures (PDB: 8YN1 and 8YL6) revealed that the ADPr‐ATP‐triggered conformational changes allow EDS1:SAG101 to interact with NRG1A (Huang et al., 2025, Xiao et al., 2025). The same two interfaces were observed, where I‐2 is completely mediated by the SAG101 EP domain and the LRR domain of NRG1A, with additional contributions by the WHD (Figure 2c). Furthermore, the mechanism was also described by which the truncated NRG1C variant (containing only the LRR and WHD motifs) inhibits signalling activation (Huang et al., 2025; Wu et al., 2022; Xiao et al., 2025). The EDS1:SAG101:NRG1C heterotrimer showed increased interactions between NRG1C and SAG101 at I‐2. NRG1C likely sequesters the EDS1:SAG101 complex, as mutations in these residues block its ability to inhibit NRG1A‐mediated ESN signalling (Huang et al., 2025, Wu et al., 2022, Xiao et al., 2025).

Both ADR1 and NRG1A can induce Ca^2+^ influx, but how they achieve distinct signalling outcomes is not well understood (Jacob et al., 2021). A recent preprint used AlphaFold 3 to predict that the NRG1A and ADR1 CC_R_ funnels are longer than those of CNLs, which may allow permeation to diverse membranes (Ibrahim et al., 2024). The authors also reported that NbADR1 localises to the plasma membrane, while NbNRG1 localises to organelles including chloroplasts, the endoplasmic reticulum and mitochondria; swapping their CC_R_ domains reversed their localisation and HR induction, suggesting a crucial role of the CC_R_ domains in determining RNL‐specific localisation and function. Organelle localisation could allow RNLs to activate immunity through yet unknown mechanism(s) additional to the established plasma membrane‐associated calcium influx.

## VARIABILITY IN THE PRODUCTION OF TIR‐DERIVED SIGNALLING MOLECULES

Plant TIR enzymatic output is complex, and it remains unknown whether all TIR domains produce the same signalling compounds, and if they are generated in similar amounts or ratios. It would be interesting to determine whether the AE interface can differentially configure the active site and contribute to enzyme variability. Within the active site, the catalytic pocket is highly conserved, particularly in the TIR domain that provides the BB loop and the required catalytic glutamate (Bayless et al., 2025; Burdett et al., 2021; Horsefield et al., 2019; Johanndrees et al., 2023; Wan et al., 2019). Despite this conservation, a TNL from *N. benthamiana* lacking the catalytic glutamate confers resistance to insects upon perception of CSPs (chemosensory proteins), suggesting that mechanisms underlying plant TIR NADase activity remain unclear (Rao et al., 2025). The DE surface, which also contributes to substrate binding, shows greater variability and is likely important for dictating enzymatic output (Bayless et al., 2025, Burdett et al., 2021, Johanndrees et al., 2023). Phosphodiester cleavage of 2’cADPR, di‐ADPR and ADPr‐ATP represent distinct chemical reactions; if and how TIR domains can carry out these reactions remains unclear.

A complete collection of the TIR domains of *A*. *thaliana* Col‐0 (the ‘AtTIRome’) has recently been assayed to explore the variation in TIR metabolite production and cell‐death signalling (Bayless et al., 2025). About half of the AtTIRome triggered cell death in transient assays in *N. benthamiana*. Artificial proteins were designed based on consensus sequences of the classes of proteins that did or did not trigger cell death, and their comparisons revealed polymorphisms that control the differences in triggering cell death and the products of NAD^+^ cleavage. The BB‐loop region was found to play a major role. The artificial TIRs were found to be functional when transplanted onto a full‐length TNL (Arabidopsis RPP1), and the BB‐loop variation could tune the activity of this protein.

## BACTERIAL TIR EFFECTORS PRODUCE 2’c ADPR AND 3’c ADPR TO PROMOTE VIRULENCE

*Pseudomonas syringae* secretes effectors through the T3SS (type three secretion system) into plant host cells to manipulate growth and immunity and promote virulence. HopAM1 is an atypical TIR effector from *P. syringae* pv. *tomato* (*Pst*) DC3000 that cleaves NAD^+^ and generates an isomer 3’cADPR (Eastman et al., 2022). Transient expression of HopAM1 in *N. benthamiana* led to NAD^+^ depletion, whereas T3SS‐mediated delivery of HopAM1 through the non‐pathogenic *Pst* DC3000 D28E mutant, which lacks 28 effector genes, did not alter NAD^+^ levels in Arabidopsis. Due to consistent 3’cADPR accumulation across these tested systems, it was suggested that 3’cADPR contributes to enhanced bacterial virulence and suppression of plant immunity. Further studies are required to determine whether and how 3’cADPR interferes with TNL‐dependent immune signalling pathways.

*P. syringae* HopBY was later identified to share structural similarity with TIR and ADPR cyclase proteins; it displays NADase activity in a catalytic glutamate‐dependent manner (Hulin et al., 2023). When HopBY was expressed in the non‐pathogenic *Pst* DC3000 D36E mutant and was delivered into Arabidopsis during infection, leaves became chlorotic and accumulated 2’cADPR, in contrast to HopAM1. While 2’cADPR is a biomarker of plant TNL immune signalling activation (Wan et al., 2019; Wang, Hu, et al., 2019), it is not sufficient to directly activate the EDS1 complexes (Bayless et al., 2023; Duxbury et al., 2020; Huang et al., 2022). It remains unclear how the same 2’cADPR molecule derived from HopBY and plant TIR enzymatic activity is distinguished and acts differentially to suppress or activate plant immunity, respectively. At the same time, enzymatic activity by HopAM1 and HopBY led to reduction of NAD^+^ levels *in planta* and triggered EDS1‐independent chlorotic cell death (Bayless et al., 2023; Choi et al., 2017; Eastman et al., 2022; Hulin et al., 2023), resembling previous observations for 2’cADPR‐producing TIR proteins from non‐phytopathogens, such as *Acinetobacter baumanii* AbTir and *Methanobrevibacter olleyae* TcpO (Bayless et al., 2023; Duxbury et al., 2020; Essuman et al., 2018; Wang, Hu, et al., 2019). It is plausible that the virulence function of these effectors may be to perturb the host NAD^+^ metabolism.

## FUNCTION OF TIR‐ONLY AND NON‐CANONICAL TIR‐CONTAINING PROTEINS

Recent findings highlight important roles for non‐canonical TIR‐containing proteins, such as C‐terminally truncated TN (TIR‐NBD) proteins, which lack an LRR domain, and TIR‐only proteins that also lack NB‐ARC domains (Meyers et al., 2002; Nandety et al., 2013). Substrate‐induced condensation was suggested as a mechanism for regulating TIR‐only proteins (Song et al., 2024). The authors showed that several TIR domains undergo phase separation *in vitro* and in plant cells when binding to NAD^+^ or ATP (Song et al., 2024). In this model, protein expression is proposed to regulate TIR function, with higher expression facilitating more TIR‐domain binding to NAD^+^ or ATP through the BE and AE interfaces. However, two recent studies of TIR‐only proteins show that inhibitory proteins keep them inactive, suggesting this may be the key aspect of regulation of non‐TNL TIR proteins, rather than relying on substrate‐induced condensation (Maruta et al., 2025; Wang et al., 2024; Wu et al., 2024). OsTIR NADase activity is inhibited through physical interactions with a sensor of Ca^2+^ and oxidative burst, ROD1 (resistance of rice to diseases 1), and during PTI and ETI, this inhibition is relieved to allow EPA formation (Wu et al., 2024). Similarly, activity of the *N*. *benthamiana* TIR‐only protein, STIR1 (stomatal TIR1), is repressed by interacting with a Ca^2+^ sensor, ISIC1 (inhibitor of stomatal immunity C2‐domain protein 1) and is de‐repressed upon PAMP treatment, enabling NADase activity and EPA formation for stomatal immunity (Wang et al., 2024).

In maize, three TIR‐only proteins were investigated for immune signalling roles. Zhang et al. (2025) demonstrated that two TIR‐only proteins, ZmTIR1 and ZmTIR3, but not ZmTIR2, induce EDS1‐dependent cell death (Zhang et al., 2025). This activity requires the catalytic glutamate in both TIR domains, and *Zmtir3* mutants have increased susceptibility to the fungal pathogen *Cochliobolus heterostrophu*s. A preprint reported that ZmTIR1 and ZmTIR2 have NADase and condensate forming activities (Kang et al., 2024). These TIR‐only proteins in maize are upregulated upon pathogen infection and potentially help to enhance immune responses. More studies on monocot and dicot TIR‐only proteins will provide insights into common and potentially different TIR signalling mechanisms and regulation.

TIR‐only proteins may have an alternative or additional signalling mechanism. Yu et al. (2022) identified that several TIR‐only proteins can bind dsDNA or RNA (Yu et al., 2022). The paper reported the cryo‐EM structure of a filament formed by flax L7^TIR^ bound to dsDNA, where the TIR domain self‐associates through the AE interface and a symmetrical DE interface (different from the BE interface found in TNL resistosomes). This binding was proposed to trigger synthetase activity that generates non‐canonical cNMPs (cyclic nucleotide monophosphates), 2′,3′‐cGMP (guanosine) and 2′,3′‐cAMP (adenosine). However, it remains unclear if this enzymatic activity is broadly conserved across plant TIR domains. In the case of the full‐length Arabidopsis TNL, SNC1, the residues found to be responsible for nucleic acid binding and synthetase activity of L7^TIR^ and several others are not required for HR (Tian et al., 2022).

Interacting partners of TN proteins have been identified and implicate these proteins in Arabidopsis immunity (Nandety et al., 2013). TN2 physically interacts with EXO7B1 (exocyst subunit exo70 family Protein B1). Pathogen‐mediated modification of EXO7B1 releases TN2, which then interacts with the calcium dependent kinase CPK5 (Huang et al., 2024; Liu, Jiang, et al., 2024; Wang et al., 2021; Zhao et al., 2015). This interaction activates the kinase and potentially triggers EPA formation. TN2 and another TN protein, CHS1 (chilling sensitive 1 or TN1), are involved in guarding the E3 ligase SAUL1 (senescence‐associated E3 ubiquitin ligase 1), a process dependent on the TNL SOC3 (suppressors of *chs1‐2, 3*) (Liang et al., 2019; Zhang, Wang, et al., 2017). A gain‐of‐function mutation in CHS1 leads to low‐temperature dependent autoimmunity, with the CHS1 mutant protein interacting with the TIR domain of SOC3. The authors suggest that TN:TNL hetero‐dimerisation activates immune signalling.

TN interactions with NLRs have also been shown for both TN10 and TN13 (Cai et al., 2021; Chen et al., 2021; Lüdke et al., 2025). The TIR domain of TN10 interacts with two TNLs, TNL40 and TNL60, although the function of this interaction remains unclear. While transient expression of TN10 does not induce cell death, it may sense a pathogen effector to modulate resistosome formation of TNL40 and TNL60 (Chen et al., 2021). Previously, TN13 and TN21 were shown to interact with the CNL RPS5, and TN13 was shown to be required to achieve RPS5‐mediated resistance to *Pst* DC3000 carrying the effector gene *avrPphB* (Cai et al., 2021). More recently, a preprint reported that TN13 also interacts with the TNL called TNT1 (TN13‐interacting TNL1) (Lüdke et al., 2025). It is possible that TN13 and TNT1 work as a pair, with TN13 potentially acting in effector recognition.

Atypical TIR‐containing proteins also exist. In cotton (*Gossypium hirsutum*), a dual TIR‐TNL (TIR‐TIR‐NBD‐LRR), GhRVD1, mediates resistance to the fungus *Verticillium dahliae* (Zhang et al., 2023). The two TIR domains require the BE interface but not necessarily the AE interface for signalling, and neither TIR domain alone can elicit cell death. Other non‐canonical TIR‐containing proteins include those with domains different from NBD and LRR domains. TIR‐PP2 (protein phloem‐2) proteins are found at least in Brassicaceae the family and contribute to mite and nematode immunity in Arabidopsis (Santamaria et al., 2019; Wojszko et al., 2023; Zuo et al., 2022). However, little is known about the functions of the TIR and PP2 domains in these proteins.

## BROADER ROLES FOR TIR DOMAIN‐MEDIATED SIGNALLING

While TIR domains have crucial roles in ETI responses, their contribution to PTI is also evident, for example in the cases of OsTIR and STIR1 (Wang et al., 2024; Wu et al., 2024). Several studies have demonstrated synergism between ETI and PTI (Tian et al., 2021; Yu, Niu, et al., 2024; Yuan, Jiang, et al., 2021; Yuan, Ngou, et al., 2021). PAMP treatments induce expression of TIR‐encoding genes, which boosts PTI responses (Tian et al., 2021). An Arabidopsis TNL, BNT1, localises to plastids and regulates PTI responses (Peppino Margutti et al., 2025). The EPA module is required for PTI (Pruitt et al., 2021). PRRs are also required for ETI responses, including the TIR‐mediated NRG1 resistosome formation (Feehan et al., 2023).

Some TIR‐containing proteins have EDS1‐independent signalling roles. The TNL SADR1 (suppressor of *ADR1‐l2 1*) partially acts in an EDS1‐independent manner to regulate gene expression and aid in pathogen containment (Jacob et al., 2023). TNPs (TIR‐NB‐ARC‐TPRs, tetratricopeptide‐like repeats) originated in land plants and are ubiquitously found across species, including those lacking the EDS1 family (Johanndrees et al., 2023; Zhang, Xue, et al., 2017). Among all tested full‐length TNPs from maize, barley, *Marchantia polymorpha* and *N. benthamiana*, only maize ZmTNP‐IIa triggers cell death independently of EDS1, and this response is dependent on the catalytic glutamate in its TIR domain. The other TNPs do not trigger HR, and the function of this class of proteins remains unknown.

## CONCLUDING REMARKS

**Figure 3:**
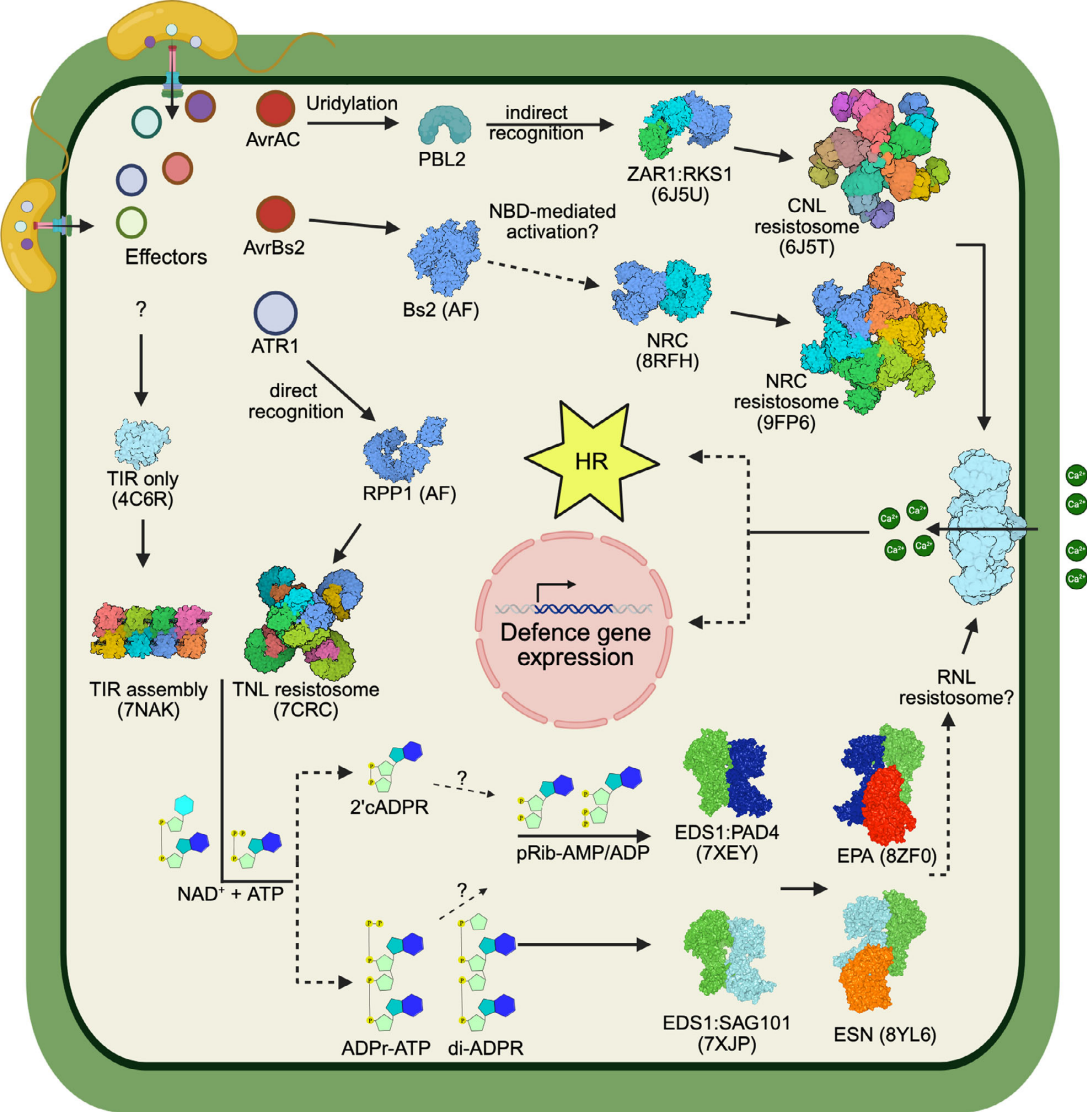
Mechanism of activation and signalling by plant NLRs. NLRs (CNLs or TNLs) directly or indirectly recognise specific effectors that are secreted by pathogenic microbes into plant cells. Effector recognition induces conformational changes in NLRs from a resting to an activated state, resulting in formation of NLR resistosomes that initiate immune signalling. Singleton NLRs, such as ZAR1 (shown as a CNL at the top), form pentameric CNL resistosomes that directly associate with the plasma membrane to act as calcium channels. In Asterids, multiple sensor CNLs (Bs2 (residues 1–855) is shown as an example, with its structure predicted using AlphaFold 3) activate downstream helper NLRs called NRCs. NRCs are homodimers in the inactive state, and effector‐induced sensor NLR activation triggers homo‐hexameric NRC resistosome assemblies, using an unknown mechanism (middle). The NRC resistosomes then serve as calcium‐conducting channels. On the other hand, the TNL‐dependent pathway (bottom) involves additional complexity. Effector‐induced activation of TNLs (AlphaFold 3‐predicted structure of RPP1 in the inactive state (residues 97–1194) is shown) results in tetrameric TNL resistosomes, where assembled TIR domains form active sites for NAD^+^ hydrolysis (in the presence of ATP). TIR‐catalysed nucleotide molecules (2’cADPR, ADPr‐ATP, di‐ADPR, pRib‐AMP/ADP) directly bind and activate the downstream heterodimers of the EDS1 family, namely EDS1:SAG101 and EDS1:PAD4. Nucleotide molecule‐bound EDS1 heterodimers can subsequently recruit helper NLRs (RNLs), forming ESN (EDS1:SAG101:NRG1) and EPA (EDS1:PAD4:ADR1) heterotrimeric complexes. Ultimately, TNL activation leads to calcium influx mediated by RNL calcium channels. Similarly, non‐NLRs, such as TIR‐only proteins, can activate EPA through NADase activity. Overall, CNL‐type NLRs all form calcium channels to induce cytoplasmic calcium influx, leading to activation of HR and transcriptional reprogramming. The figure was generated using BioRender.

Upon pathogen attack, formation of NLR resistosomes in their distinct oligomeric configuration is key to activation of an initial step of ETI signalling (Figure 3; Box 1). Resistosomes formed by CNLs, NRCs and TNL‐induced RNLs all serve as calcium ion channels, triggering cytoplasmic calcium influx. What comes after NLR‐mediated calcium influx remains unclear. Calcium acts as a second messenger to activate multiple downstream signalling cascades, to induce reprogramming of shared sets of genes during PTI and ETI, and/or to elicit cell death directly due to its constitutive elevation during ETI (Kim et al., 2022).

Box 1 **Summary**

- Plant NLRs recognise pathogen effector proteins through direct and indirect mechanisms and assemble into resistosomes.
- NLRs can function alone or as pairs, or they rely on helper NLRs to execute immune signalling.
- CC‐containing NLRs, including CNLs and helper CC_R_‐NLRs, initiate immune response by forming calcium channels to trigger Ca^2+^ influx.
- Stoichiometries of CNL resistosomes differ, depending on subclades; CC‐NLRs form pentamers, Asterid‐specific NRCs form hexamers and CC_G10_‐NLRs form octamers.
- TIR‐containing NLRs or TIR‐only proteins generate second messenger signalling molecules upon cleavage of NAD^+^, to recruit EDS1:SAG101:NRG1 or EDS1:PAD4:ADR1 modules.

While we now understand that TIR‐generated products selectively recruit EPA and ESN modules, whether the ESN and EPA heterotrimers serve as precursors to RNL calcium channels remains unknown, with the lack of structural information on RNL resistosomes. It is also unclear if EDS1:PAD4 and EDS1:SAG101 complexes are components of the RNL resistosome or must dissociate prior to their formation. Two glutamate receptor‐like calcium ion channels (GLR2.9a and b) have been recently identified to operate specifically upon activation of the TNL‐dependent ESN pathway in *N. benthamiana*, contributing to HR and disease resistance (Wang, Sun, et al., 2025). Further studies will help to dissect ETI signalling pathways (Box 2).

Box 2 **Open Questions**

- How do sensor NLRs induce executor NRCs to form homo‐oligomeric resistosomes?
- What effect does the pore size of CNL calcium channels have on immune responses?
- Upon activation, how do paired TNLs associate to create an NAD^+^ binding site between two TIR domains?
- Does variability in plant TIR enzyme output lead to differential recruitment of EDS1 signalling modules?
- How do EPA and ESN heterotrimers lead to RNL resistosomes and what oligomeric state do these form?

Available plant NLR structures in the resting state are currently limited to CNLs (ZAR1 and NRC2); the two exhibit different oligomeric forms. There is need for further research into various classes of inactive NLR structures to elucidate their autoinhibitory mechanisms. Structural studies of paired TNLs and CNLs will also enhance our understanding of the mechanisms that regulate their oligomerisation and signalling. Structure prediction tools can complement structural biology techniques. AlphaFold 3 was used to model, with high confidence, regions that are challenging to resolve experimentally, such as the N‐terminal α1 helices of membrane‐associated CNL resistosomes, by including lipids in the predictions (Madhuprakash et al., 2024). AlphaFold predictions can also reveal characteristic patterns and help categorise NLRs, such as the sensor or helper NLR types, guiding researchers in prioritising candidates for further functional and structural investigation (Toghani et al., 2025). While AlphaFold structure prediction can aid NLR research significantly, its limitation in handling large amino acid sequences precludes analysis of full‐length NLR resistosomes with varying stoichiometries. Therefore, experimental structure determination and functional validation remain essential (Box 2). Pathogen‐derived effectors that activate plant NLRs often show limited sequence similarities with any characterised proteins; therefore, structure prediction of these proteins often remains challenging (Jones & Raffaele, 2025; Maruta, Outram, & Kobe, 2023).

Understanding how NLRs sense effectors and transition from inactive and intermediate to active forms enables the development of strategies for engineering novel immune receptors to combat plant diseases. Effector recognition capabilities can be expanded by modifying LRR domains or by adding or substituting integrated decoy domains, which have been extensively studied in CNLs (Zdrzałek et al., 2023). As an example, based on findings from the Sr35 cryo‐EM structure, LRR domains from NLRs of unknown defence function in barley and wheat were replaced with the Sr35 LRR domain (Förderer et al., 2022). These hybrid NLRs were introduced into a susceptible wheat cultivar, which gained new recognition specificity for the effector from the highly aggressive stripe rust fungus *P. graminis* f. sp. *tritici* (Förderer et al., 2022). Another approach involves engineering chimeric NLRs with N‐terminal regions cleavable by pathogen‐derived proteases, connected to autoactive CNLs or RNLs; these chimeras were shown to trigger disease resistance to a broad spectrum of viruses (Wang, Chen, et al., 2025). Tuning the enzymatic output of TIR domains could be another promising strategy (Bayless et al., 2025). Typically, ESN triggers a strong, localised cell‐death response, while EPA contributes to disease resistance and transcriptional reprogramming (Bhandari et al., 2019; Dong et al., 2016; Lapin et al., 2019; Lapin et al., 2020; Wu et al., 2019). Altering TIR enzyme output may lead to differential recruitment of either module, potentially allowing plants to tailor immune responses to different pathogen classes and across diverse species. Whether certain TIR protein architectures confer a fitness advantage over others is also an intriguing concept and could help prioritise which TIR proteins to engineer.

## CONFLICT OF INTEREST STATEMENT

The authors declare no conflicts of interest.

## Data Availability

Data sharing is not applicable to this article as no datasets were generated or analysed during the current study.
